# Continuous Physiological Monitoring of the Combined Exposure to Hypoxia and High Cognitive Load in Military Personnel

**DOI:** 10.3390/biology12111398

**Published:** 2023-11-03

**Authors:** Leonard A. Temme, Harrison L. Wittels, Michael J. Wishon, Paul St. Onge, Samantha M. McDonald, Dustin Hecocks, S. Howard Wittels

**Affiliations:** 1 Army Aeromedical Research Laboratory, Fort Novosel, AL 36362, USA; leonard.a.temme.civ@health.mil (L.A.T.); paul.m.stonge2.civ@health.mil (P.S.O.); 2 Tiger Tech Solutions, Inc., Miami, FL 33140, USAjoe@tigertech.solutions (M.J.W.); dustin@tigertech.solutions (D.H.); shwittels@gmail.com (S.H.W.); 3 School of Kinesiology and Recreation, Illinois State University, Normal, IL 61761, USA; 4 Department of Anesthesiology, Mount Sinai Medical Center, Miami, FL 33140, USA; 5 Department of Anesthesiology, Wertheim School of Medicine, Florida International University, Miami, FL 33199, USA; 6 Miami Beach Anesthesiology Associates, Miami, FL 33140, USA

**Keywords:** hypoxia, hyperoxia, aviation, military, autonomic nervous system, heart rate variability, sympathetic nervous system

## Abstract

**Simple Summary:**

The health and safety of military aviators is paramount during flight operations. In flight, aviators experience extreme environmental conditions such as high altitude, which reduces oxygen availability to the brain and compromises the function of all bodily systems. The autonomic nervous system (ANS) regulates many of the bodily systems, and therefore its function is a strong indicator of the physiological consequences to prolonged exposure to less oxygen. Importantly, aviators spend most of their flight time at less severe altitudes. However, even mild decrements in oxygen may elicit suboptimal function of the ANS, compromising aviator safety. What remains less clear is how the exposure to mild reductions in oxygen while simultaneously performing simulated flight tasks affects the ANS. The current study investigated this question by exposing aviators to varying levels of oxygen while carrying out simulated flight tasks. The aviators’ ANS responses were measured throughout the nearly two hours of trials. Our study observed heightened sympathetic nervous system activity (e.g., “fight or flight”) and found suggestions of increased anxiety. Lastly, we found that the timing and extent of the ANS responses differed between conditions. These observations highlight the importance of monitoring several markers of ANS function to avoid deteriorating aviator function when flying at mild altitudes.

**Abstract:**

Military aviators endure high cognitive loads and hypoxic environments during flight operations, impacting the autonomic nervous system (ANS). The synergistic effects of these exposures on the ANS, however, are less clear. This study investigated the simultaneous effects of mild hypoxia and high cognitive load on the ANS in military personnel. This study employed a two-factor experimental design. Twenty-four healthy participants aged between 19 and 45 years were exposed to mild hypoxia (14.0% O_2_), normoxia (21.0% O_2_), and hyperoxia (33.0% O_2_). During each epoch (*n* = 5), participants continuously performed one 15 min and one 10 min series of simulated, in-flight tasks separated by 1 min of rest. Exposure sequences (hypoxia–normoxia and normoxia–hyperoxia) were separated by a 60 min break. Heart rate (HR), heart rate variability (HRV), and O_2_ saturation (SpO_2_) were continuously measured via an armband monitor (Warfighter Monitor^TM^, Tiger Tech Solutions, Inc., Miami, FL, USA). Paired and independent *t*-tests were used to evaluate differences in HR, HRV, and SpO_2_ within and between exposure sequences. Survival analyses were performed to assess the timing and magnitude of the ANS responses. Sympathetic nervous system (SNS) activity during hypoxia was highest in epoch 1 (HR: +6.9 bpm, *p* = 0.002; rMSSD: −9.7 ms, *p* = 0.003; SDNN: −11.3 ms, *p* = 0.003; SpO_2_: −8.4%, *p* < 0.0000) and appeared to slightly decline with non-significant increases in HRV. During normoxia, SNS activity was heightened, albeit non-significantly, in epoch 1, with higher HR (68.5 bpm vs. 73.0 bpm, *p* = 0.06), lower HRV (rMSSD: 45.1 ms vs. 38.7 ms, *p* = 0.09 and SDNN: 52.5 ms vs. 45.1 ms, *p* = 0.08), and lower SpO_2_ (−0.7% *p* = 0.05). In epochs 2–4, HR, HRV, and SpO_2_ trended towards baseline values. Significant between-group differences in HR, HRV, and O_2_ saturation were observed. Hypoxia elicited significantly greater HRs (+5.0, *p* = 0.03), lower rMSSD (−7.1, *p* = 0.03), lower SDNN (−8.2, *p* = 0.03), and lower SpO_2_ (−1.4%, *p* = 0.002) compared to normoxia. Hyperoxia appeared to augment the parasympathetic reactivation reflected by significantly lower HR, in addition to higher HRV and O_2_ relative to normoxia. Hypoxia induced a greater ANS response in military personnel during the simultaneous exposure to high cognitive load. The significant and differential ANS responses to varying O_2_ levels and high cognitive load observed highlight the importance of continuously monitoring multiple physiological parameters during flight operations.

## 1. Introduction

Relative to all other bodily tissues, the human brain consumes the largest amount of oxygen (O_2_) [1], as it regulates all physiological and cognitive processes. Cerebral O_2_ demand rises with the increasing complexity of these processes [1]. Processes such as higher-order cognitive functions require more energy and thus a greater supply of O_2_ [2,3,4]. Sufficient O_2_ availability becomes critical for individuals like military aircrew who endure high cognitive loads during flight operations, including executing life-or-death decisions within infinitesimal timeframes [5,6]. Importantly, while flying, military aircrew are exposed to high-altitude environments (>8000 ft), which compromises O_2_ availability due to the decreasing partial pressure of O_2_ (PO_2_) in atmospheric air [7]. Correcting for this O_2_ deficit requires the autonomic nervous system (ANS) to respond rapidly and sufficiently to preserve the integrity of their cognitive function.

Many previous studies evaluated the physiological responses to hypoxic environments among military aircrew, and expectedly showed a disruption in cardiorespiratory function [8]. Specifically, aircrew members experienced increased heart rates (HRs), decreased arterial O_2_ saturation, depressed HR variability (HRV), and hyperventilation [9]. The observed sympathetic dominance of the ANS demonstrates the prioritization of increasing cerebral O_2_ availability. These physiological responses are well established at extremely high altitudes that present a severely hypoxic exposure [10]. Here, the ANS response cannot sustain an adequate supply of O_2_ to maintain brain function beyond 120 to 180 s [7,11]. However, during most flight excursions, military aircrew spend more time flying at lower altitudes (8000–10,000 ft), and thus are exposed to milder levels of hypoxia. Scientific evidence for the effects of mild hypoxia on ANS response and function is equivocal, with some studies showing significant alterations in cardiorespiratory function and others reporting no effects [7,12]. The ambiguous evidence may be attributable to the study design of former studies. Many studies examined the influence of mild hypoxia on the response of the ANS; however, during the hypoxic exposure, military aircrew were either performing tasks with low cognitive loads [13,14] (e.g., memorizing, learning basic skill) or resting (e.g., seated quietly). While important, these scenarios do not accurately reflect the high cognitive load aircrew frequently endure [15].

Understanding the nature of the ANS response to mild hypoxia while simultaneously performing simulated, highly cognitive tasks often occurring during flight is paramount to the safety of military aircrew. Moreover, because aircrew fluctuate between varying levels of high altitude, also understanding the ANS response to hyperoxia may be important, especially for potential solutions following severe hypoxic exposure [8]. Currently, to our knowledge, no previous studies evaluated the simultaneous effects of mild hypoxia and high cognitive loads on the ANS. Specifically, this study investigated the magnitude and time interval of the initial and subsequent responses of the ANS of aircrew to mild hypoxia (O_2_ = 14.0%) while continuously performing simulated, in-flight tasks for 125 min (about 2 h).

## 2. Materials and Methods

### 2.1. Study Design and Sample

This study was part of a larger study that employed a mixed-model, within-subject, two-factor (A and B) experimental design. All subjects were exposed to the two levels of Factor A and the 5 levels of Factor B. The two Factor A levels were the schedule of breathable gases, with each schedule lasting 134 min. One Factor A schedule consisted of breathing 14% O_2_ (normobaric–hypoxic) continuously for 108 min immediately followed by breathing 21% O_2_ (normobaric–normoxic) for 26 min. The other Factor A schedule consisted of breathing 21% O_2_ (normobaric–normoxic) continuously for 108 min immediately followed by breathing 33% O_2_ (normobaric–hyperoxic) for 26 min. Subjects were tested with one schedule in the morning and the other schedule in the afternoon, following lunch. The schedule tested first alternated between subjects, so that the accrual of data was counterbalanced.

Factor B was a pair of computer-generated surrogate flight-relevant tasks that subjects repeatedly performed throughout the two 134-min periods of testing. The 134-min testing direction was divided into 5 successive epochs to evaluate the effects of prolonged exposure.

### 2.2. Subjects

The 24 volunteer subjects were a self-selected sample drawn from the local geographic area around Fort Novosel, AL, USA. The sample consisted of military and civilian personnel recruited via study fliers, email, social media, word of mouth, etc. Individuals interested in the study contacted the advertised research team point of contact, who prescreened volunteers for eligibility. Participants were eligible for the study if they were (1) between 19 and 45 years of age, (2) in an “off-duty” status, (3) not pregnant, and (4) reported no history of amblyopia, strabismus, corrective eye muscle surgery, or severe altitude-related illness. Participants were excluded from the study if they (1) exhibited symptoms of respiratory or sinus infection or flu, (2) used tobacco products in the last 6 months, (3) donated blood in the last 30 days, (4) spent 10 or more days at an altitude greater than 5000 ft above mean sea level in the past 3 months, (5) reported a history of excessive alcohol use during the last 6 months, and (6) were not under medical treatment for psychiatric, neurological or sleep-related problems, anemia, asthma, heart/circulatory disease, hypertension, sickle cell anemia, emphysema, seizure disorder, chronic stress, concussion with loss of consciousness, or attention deficit hyperactivity disorder. All prospective participants were fully informed of the study protocol, measurements, risks, and benefits in accordance with the Declaration of Helsinki. All subjects voluntarily consented to participate in the study. By agreement with the subjects, the gender and ethical attributes of the subjects cannot be disclosed. The protocol and study procedures were approved by the Headquarters, U.S. Army Medical Research and Development Command Institutional Review Board (HQ USAMRDC IRB M-10859; approval date 28 December 2020).

### 2.3. O_2_ Exposures (Factor A)

The three pre-mixed compressed air gas combinations were obtained from a commercial supplier (Airgas, Air Liquide Company, Radnor Township, PA, USA). These pre-mixed combinations were (a) 21.0% O_2_ balanced with 79.0% N to emulate mean sea level (MSL) air, the normoxia condition; (b) 14.0% O_2_ balanced with 86.0% N, which approximates the O_2_ content of air typically encountered at about 10,000 feet above MSL, the hypoxia condition; and (c) 33.0% O_2_ balanced with 67.0% N, to provide a moderately O_2_-enriched hyperoxic condition.

These gases were delivered to a subject via an aviator Mask Breathing Unit (MBU-20/P, Gentex Corp., Zeeland, MI, USA) commonly used by high-performance fighter-jet pilots. The mask is held in place over the oronasal cavity with an aviator’s helmet. Each volunteer was individually fit with the appropriately sized helmet and mask to maintain a secure comfortable seal for the duration of the study. The gas was delivered to the mask via a Cobham 29,270 oxygen regulator CRU-73 automatic diluter-demand pressure breathing oxygen regulator designed for use in high-performance aircraft. A built-in pressure reducer regulated the inlet supply pressure to 50 psig (344.7 kPa). Breathing gas was delivered to the mask by means of a flexible hose attached to the outlet of the regulator.

### 2.4. Simulated In-Flight Task Exposure (Factor B)

The cognitive load to which the subjects were exposed consisted of the extended performance of a pair of aviation-relevant computerized task batteries, the Automated Desktop Vision Tester (ADVT) [16] and the Enhanced Air Force Multi-Attribute Task Battery (EAF-MATB) [17,18]. Each ADVT testing required 15 min to complete and consisted of a set of computer-based vision tests that included measurements of conventional static stereo acuity, dynamic stereo acuity, two-dimensional tracking, as well as horizontal and vertical fusion range determinations. Each EAF-MATB testing required 10 min to complete and consisted of a set of computer-based surrogate aviation tasks such as visual target tracking, auditory and visual signal monitoring, resource management, and responses to event onsets. The specific tests administered include the following: Conventional Stereo Acuity Near Threshold (CSA N.T.), Conventional Stereo Acuity Far Threshold (CSA F.T.), Dynamic Stereo Acuity (D.S.A.), 2D Positional Tracking (2D P.T.) and Fusional Range (F.R.).

**Figure 1:**
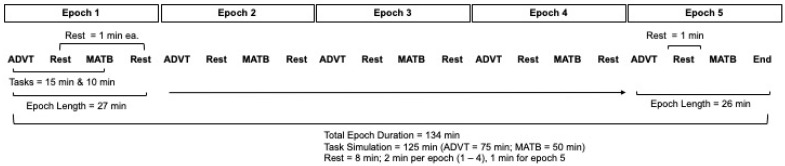
Schematic of the Cognitive Load Testing.

For each of the two Factor A gas breathing conditions, performance of the ADVT and EAF-MATB tasks was successively alternated five times to generate a total of 125 min of continuous performance testing, with an additional 1 min allocated for personnel to transition from one task to the other. With 9 such task transitions, the total exposure for each Factor A condition was 134 min (see Figure 1). Thus, the 134 min was divided into 5 epochs. The first 4 epochs each consisted of 15 min ADVT testing, 1 min transition for 10 min EAF-MATB testing, the 1 min transition for 15 min ADVT testing, and so forth for 4 iterations. The 5th epoch did not include the final 1 min transition since the trial terminated.

### 2.5. Hypoxic–Normoxic (H-N) Condition

For the H-N condition, subjects repeatedly performed the ADVT and EAF-MATB tasks while exposed to 14% O_2_ for 108 min of epoch 1 through 4. Importantly, at the start of the 5th epoch the hypoxic gas was precipitously switched to the normoxic gas (21.0% O_2_) for the final 26 min.

### 2.6. Normoxic–Hyperoxic (N-Hyper) Condition

For the N-Hyper condition, subjects repeatedly performed the ADVT and EAF-MATB tasks while exposed to 21.0% O_2_ for 108 min of epochs 1 through 4. Importantly, at the start of the 5th epoch, the normoxic gas was precipitously switched to the hyperoxic gas (33.0% O_2_) for the final 26 min. Note that a 1 h lunch break divided morning from afternoon testing.

### 2.7. Autonomic Nervous System Response (Outcome)

The responses of the ANS to normoxia, hypoxia, hyperoxia, and cognitive load were measured via an armband with electrocardiographic and pulse oximetry capabilities (Warfighter Monitor^TM^ (WFM), Tiger Tech Solutions, Inc., Miami, FL, USA), which were previously validated in diverse populations [19]. Subjects wore the armband on the posterior aspect of their upper right arm, and it was fitted around the widest part of the biceps with an elastic band. HR and HRV were used as strong indicators of ANS activity. HR is defined as the number of times the cardiac muscle contracts during a 60 s interval (beats per min; bpm). HRV is defined as the time variation between heartbeats. The metrics used to evaluate HRV included the standard deviation of NN intervals (SDNN) and the root mean square of successive differences (rMSSD), described in detail elsewhere [20]. HR and HRV (2 min norm) were continuously measured throughout each experimental trial except during the 60 min break between trials. Pulse oximetry was continuously measured (SpO_2_) to estimate the level of arterial O_2_ saturation in the blood [21,22].

### 2.8. Statistical Analysis

All groups were tested for normality using Kolmogorov–Smirnov testing and were found to be normally distributed. Means, standard deviations, and line plots were calculated for all groups. Survival analysis was performed with the time-to-event threshold defined as an increase or decrease of at least 5 units for all outcomes of interest. No data were censored. Within- and between-group differences were performed via paired and independent *t*-tests to evaluate the differences in ANS responses to the simultaneous effects of hypoxia, normoxia, or hyperoxia and high cognitive load. Specifically, within-group comparisons examined the changes in HR, HRV, and SpO_2_ within individuals exposed to hypoxia across epochs 1–4 and then normoxia in epoch 5. The same procedure was applied to the normoxia–hyperoxia sequence (Table 1). Differences in HR, HRV, and SpO_2_ between the exposure sequences (hypoxia–normoxia vs. normoxia–hyperoxia at each epoch were evaluated (Table 2). Statistical analysis was performed using MATLAB R2022a. Statistical significance was set a priori at α < 0.05.

## 3. Results

Twenty-four military and civilian personnel participated in the current study. Subjects were apparently healthy, aged between 19 and 45 years of age, and did not exhibit any cardiovascular, neurological, respiratory, cerebral, or metabolic abnormalities or chronic conditions. No adverse events were reported during the study period.

**Figure 2:**
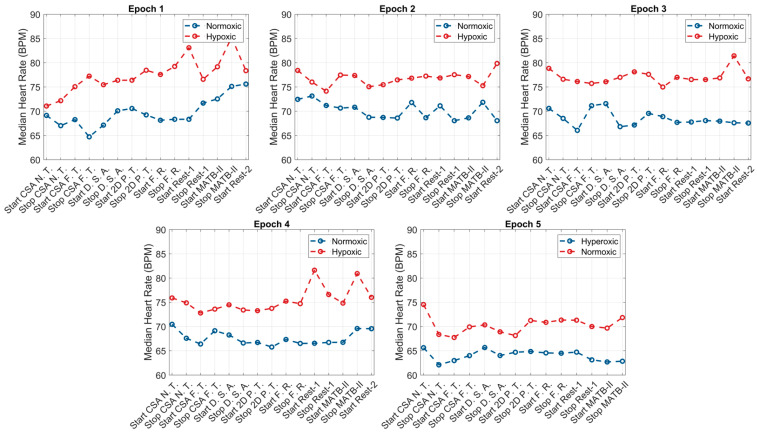
HR responses to the simultaneous effects of hypoxia, normoxia, or hyperoxia and high cognitive load across all epochs.

**Figure 3:**
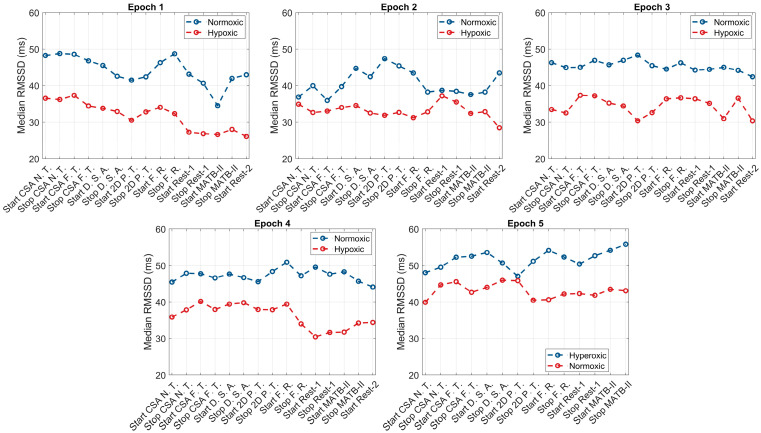
rMSSD responses to the simultaneous effects of hypoxia, normoxia, or hyperoxia and high cognitive load across all epochs.

**Figure 4:**
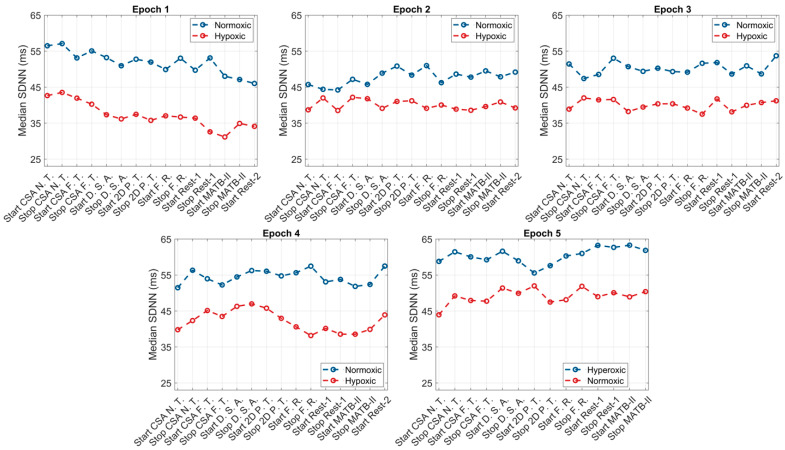
SDNN responses to the simultaneous effects of hypoxia, normoxia, or hyperoxia and high cognitive load across all epochs.

The within-group responses of the ANS to hypoxic, normoxic, and hyperoxic exposures are presented in Table 1. For the hypoxic condition, across the first four epochs (108 min), individuals exhibited higher HRs and lower HRV (both rMSSD and SDNN) and O_2_ saturation levels compared to their baseline values. The greatest increase in SNS activity occurred in and was sustained throughout epoch 1, reaching peak values at the start of the second epoch (HR: +6.9 bpm, *p* = 0.0002; rMSSD: −9.1). Throughout the second, third, and fourth epochs, SNS activity appeared to slightly decline with non-significant increases in HRV (rMSSD: +0.2 ms, +1.2 ms, and +3.2 ms; SDNN: +0.2 ms, +1.4 ms, and +3.7 ms). Upon exposure to normoxia (epoch 5), non-significant decreases in HR (−2.3 bpm, *p* = 0.13), non-significant increases in HRV (rMSSD: +5.2 ms, *p* = 0.16 and SDNN: +6.1 ms, *p* = 0.16), and significant increases in O_2_ saturation (SpO_2_: +8.9%, *p* < 0.0001) were observed. At the end of the normoxic trial, subjects elicited non-significant differences in HR (−0.1 bpm, *p* = 0.67) and HRV (rMSSD: +0.1 ms, *p* = 0.74 and SDNN: +0.1 ms, *p* = 0.78) and significantly higher O_2_ saturation (+1.1%, *p* = 0.0003) compared to baseline values. Graphical representations of these results are presented in Figure 2, Figure 3 and Figure 4.

Like the hypoxic condition, the SNS activity of subjects during normoxia was heightened during epoch 1, with increases in HR (68.5 bpm vs. 73.0 bpm, *p* = 0.06), decreases in HRV (rMSSD: 45.1 ms vs. 38.7 ms, *p* = 0.09 and SDNN: 52.5 ms vs. 45.1 ms, *p* = 0.08), and decreases in SpO_2_ (−0.7% *p* = 0.05). At the start of epoch 2, HR (73.0 bpm vs. 71.3 bpm vs. 71.0 bpm), rMSSD (38.7 ms vs. 41.1 ms vs. 41.5 ms), SDNN (45.1 ms vs. 47.9 ms vs. 48.3), and SpO_2_ trended towards baseline values and continued through epochs 3 and 4. However, these changes did not reach the a priori level of statistical significance. Exposure to hyperoxia, occurring during epoch 5, appeared to augment parasympathetic dominance, reflected by further non-significant reductions in HR (−3.8 bpm, *p* = 0.24), non-significant increases in HRV (rMSSD: +5.3 ms, *p* = 0.26 and SDNN: +6.1 ms, *p* = 0.26), and significant increases in O_2_ saturation (+1.3%, *p* = 0.003). Relative to baseline, subjects elicited lower HRs and higher HRV and O_2_ saturation following the hyperoxic trial; however, these differences did not reach statistical significance. Graphical representations of these results are presented in Figure 2, Figure 3 and Figure 4.

Table 2 presents the between-group differences in the ANS response to the simulated, in-flight tasks between the hypoxic, normoxic, and hyperoxic exposures. Throughout epochs 1–4, statistically significant differences in HR, HRV, and O_2_ saturation were observed between the hypoxic and normoxic exposures. Subjects exposed to hypoxia elicited significantly greater HRs (+5.0, *p* = 0.03), lower rMSSD (−7.1, *p* = 0.03), lower SDNN (−8.2, *p* = 0.03), and lower SpO_2_ (−1.4%, *p* = 0.002) compared to the normoxia condition. Additionally, the magnitude of differences in ANS response between hypoxia and normoxia appeared consistent throughout the 108 min trial, with the largest differences observed in the third epoch. During epoch 5, hyperoxia appeared to augment the parasympathetic reactivation, yielding significantly lower HRs and higher HRV and SpO_2_ relative to the normoxia that followed a hypoxia exposure.

The magnitude and rate of SNS activity in response to hypoxia and in-flight, simulated tasks are presented in Figure 4. By 60 s into the first epoch, 100% of subjects exposed to hypoxia elicited at least a 5% decrease in O_2_. For the cardiac measures, 100% of the sample exhibited a 5 bpm increase in HR and a 5 ms and 5% decrease in HRV and O_2_, respectively, nearly four minutes into the trial. At the onset of normoxia (epoch 5), 100% of the sample exhibited at least a 5 bpm decrease in HR and a 5 ms and 5% increase in HRV and O_2_ saturation, respectively. These results indicate that O_2_ saturation responds fastest to hypoxic and normoxic exposures, followed by HR and HRV.

## 4. Discussion

This study investigated the simultaneous effects of mild hypoxia on the ANS among individuals continuously performing aviation-relevant tasks for 125 min. The major findings of this study were severalfold. First, within the first few minutes, all volunteers exposed to the hypoxic condition exhibited sympathetic response, as reflected by higher HR and lower HRV indices. Interestingly, it appeared that the magnitude of the ANS response was influenced by the order of the hypoxic–normoxic exposure. Volunteers tested first under mild hypoxia, i.e., in the morning, exhibited higher HRs compared to volunteers tested first under normoxia. Second, after ~30 min the ANS exhibited potential adaptations to the hypoxic exposure, as shown by decreased yet still elevated HRs and less depressed values for HRV. Third, within 4 min of normoxia following hypoxia (epoch 5), HR and HRV values returned to baseline for all volunteers. Lastly, following the normoxic condition, the hyperoxic exposure (epoch 5) appeared to augment parasympathetic dominance, as evidenced by further reductions in HR and increases in HRV values.

### 4.1. ANS Response to Acute Hypoxia

The ANS responses to mild hypoxia observed in this study expanded upon the existing scientific evidence. The hypoxia-induced sympathetic dominance observed is consistent with previous hypoxia studies [7,8]. Specifically, when exposed to hypoxia, the participants exhibited expected increases in HR and decreases in HRV that were significantly different than those during normoxia across all epochs. This observation is supported by well-documented physiological evidence. Former studies established that in response to lower arterial PO_2_, the body prioritizes increasing O_2_ supply to the brain through processes such as augmenting cardiac output and vasodilation of cerebral arteries [9,23,24]. These immediate responses result in heightened HR and depressed HRV values, as demonstrated in the current study. One unique aspect of our findings, however, was quantifying the magnitude and timing of the initial response of the ANS to the mild hypoxic exposure. After 90 s of hypoxic exposure, 50% of the subjects showed a ≥5 bpm increase in HR, whereas only 5% showed such an increase under normoxia. By 3.5 min into the hypoxic exposure, 100% of the subjects showed a ≥5 bpm change, compared to only 35% while normoxic. Although a higher ANS response while hypoxic relative to normoxic was expected, the large differences observed in magnitude and timing were unanticipated given the mild level of hypoxia (14% O_2_). Previous studies evaluating the pre- and post-exposure responses of the ANS to hypoxia demonstrated dose-dependent effects, with large effects shown mostly at severe levels of hypoxia (≤10% O_2_) [25,26,27]. Interestingly, evidence shows that aircrew members exposed to mild-to-moderate levels of hypoxia self-reported several known hypoxia-induced symptoms like fatigue, headache, nausea, and dizziness at milder levels of hypoxia (12–15% O_2_) [28,29,30]. Collectively, these and our findings may indicate that aircrew flying at 10,000 ft are, to a concerning level, immediately and negatively affected by mild hypoxic exposure. Given the constant life-or-death decision making performed by aircrew, providing supplemental O_2_ at lower altitudes may be advantageous for the safety of aircrew.

### 4.2. Influence of High Cognitive Load on ANS Response to Acute Hypoxia

A novel aspect of the above findings was that these hypoxia-induced effects on the ANS occurred in the presence of an additional stressor. During both normoxia and hypoxia, high cognitive loads were imposed across all epochs. To effectively execute demanding cognitive tasks, the brain requires additional energy and thus a greater O_2_ supply for optimal performance [3,4]. Findings from the current study corroborate this notion; upon initial exposure to the cognitive tasks, normoxic participants exhibited a small, transient increase in HR with a reduction in HRV. This response reflects the activation of the SNS, which leads to physiological processes that increase the energy supply to the brain (e.g., gluconeogenesis and glycogenolysis) [31,32]. Importantly, when confronting the same high cognitive load when hypoxic, the differences in HR and HRV were unexpectedly large compared to the normoxic response. Notably, experiencing these exposures simultaneously realistically reflects the operational demands aircrew routinely encounter while flying.

Although many studies previously evaluated the effects of hypoxia on cognitive performance, of which several found a dose–response impairment, the participants did not continuously experience both exposures [5,13,14,28,33]. Often, former studies provide either brief or prolonged hypoxic exposures followed by a single assessment or series of cognitive assessments. Demonstrating hypoxia-induced cognitive impairments provides critical information regarding the aircrew’s ability to effectively perform in-flight operations. The study designs previously employed, however, assume aircrew endure high cognitive loads intermittently rather than continuously, which does not accurately reflect the demands of their environment. Moreover, a vast majority of these studies evaluated cognitive capacity at more severe levels of hypoxia. Thus, the additional physiological demand of performing high-cognitive-load tasks may induce larger, more negative effects than those previously reported. Therefore, it is crucial that the cognitive demand of flight tasks is accounted for in addition to the physiological effects induced by hypoxia.

### 4.3. Influence of Anxiety on the ANS Response to Hypoxia and Cognitive Load

Interestingly, our study also showed that anxiety may contribute to the heightened ANS response to mild hypoxia. As previously stated, hypoxia elicited large and rapid increases in HR and HRV when initially exposed to mild hypoxia relative to normoxia. The hypoxic-induced changes in ANS function, however, appeared to depend upon the order in which the participants received the hypoxic condition. Specifically, the participants randomized to the hypoxic–normoxic group experienced larger increases in HR and HRV compared to their counterparts in the normoxic–hypoxic group. Substantial evidence demonstrates that reduced O_2_ availability activates the hypothalamus–pituitary–adrenal axis, specifically the amygdala, which strongly influences such emotional responses as anxiety [34]. Studies previously demonstrated higher emotional reactivity (e.g., anxiety and agitation) among individuals exposed to hypoxia, which activates the SNS and elicits similar effects on HR and HRV [35,36,37]. Lower PO_2_ resembles feelings of suffocation, one of the strongest triggers of the fight-or-flight response [38]. Thus, it is possible that following the immediate exposure to 14% O_2_, the participants, specifically those in the hypoxic–normoxic group, experienced a certain level of anxiety, potentially exacerbating their ANS response. For the normoxic–hypoxic group, we speculate that the lower ANS response to hypoxia may be attributed to a few extraneous factors. First, while the participants were unaware to which condition they were randomized for the first trial, it is possible that the participants exposed to hypoxia in the second trial were more prepared for the abrupt change in O_2_ availability, and thus were able to tolerate any associated anxiety. Second, by the second trial, the normoxic–hypoxic group was familiarized with several aspects of the protocol, like the surrounding environment, wearing the oxygen mask, and performing the simulated in-flight tasks. Both the beforehand knowledge and familiarity with the protocol potentially explain the lower increase in HR and decrease in HRV response to mild hypoxia for the normoxic–hypoxic group relative to the hypoxic–normoxic group. The possible anxiety-provoking effects of mild hypoxia exposure demonstrated in the current study highlight the importance of (1) training aircrew on effective strategies for managing the onset of anxiety symptoms and (2) repeated exposure to hypoxic conditions to familiarize aircrew with the physiological responses. Other studies found that prior exposure to hypoxic conditions reduced the magnitude of the associated effects on the SNS [34,39,40]. Moreover, studies observed that pilots who participated in intermittent hypoxia training more quickly identified the onset of hypoxia-induced symptoms and initiated subsequent emergency procedures [41]. What remains less clear, however, is the most effective program for intermittent hypoxia training regarding the frequency (i.e., daily, weekly, or monthly), severity of hypoxia, duration of exposure, timing prior to flight assignments, shelf life of physiological adaptations, etc.

### 4.4. ANS Adaptation to High Cognitive Load and Hypoxia

Another major finding of this study was the observed reduction in the SNS response: nearly 30 min into the hypoxic and normoxic trials. This contrasts former studies that reported a plateauing effect or a persistent increase in SNS activity in response to hypoxia. The latter finding was often reported for studies exposing participants to more severe hypoxic conditions [27,42]. The decrease in SNS activity observed in the current study is likely an adaptation to a few different factors. First, given that a similar reduction in SNS activity occurred during the normoxic condition, it is possible that the decline was attributed to “settling in.” The exposure to a high cognitive load likely contributed, in part, to the initial increase in SNS activity during normoxia, and, as previously mentioned, possibly anxiety. With that, we speculate that the decline in SNS activity occurred because the participants adjusted to their physical environment [43,44]. The subjects were possibly less stimulated by the fitted oxygen mask, wearing several measurement devices, and learning the logistics of completing the various tasks. In support of this speculation, both HR and HRV during normoxia trended toward a parasympathetic reactivation throughout the remainder of the trial (epochs 2–4). Conversely, during hypoxia, HR and HRV in epochs 2 through 4 remained elevated and depressed, respectively. These values, however, were lower than the maximum value reached at the end of the first epoch. Like the normoxic group, the hypoxic group was exposed to a high cognitive load, and as such, the decline observed during hypoxia may also be attributed to familiarization. However, it is highly likely that the decrease in SNS activity was due to participants adapting physiologically to the hypoxic exposure. It is well documented that the human body drastically alters physiological processes in the presence of hypoxia to preserve brain function by increasing O_2_ availability. Interestingly though, unlike other studies, a plateau in SNS activity while exposed to mild hypoxia was not achieved [33,42]. In fact, the values were trending downward. Differences in the experimental designs between studies potentially explain this observation. In the current study, the participants were continuously exposed to a constant, high cognitive load in addition to hypoxia. Other studies tested the subjects’ performance on simulated in-flight operations while hypoxic; however, the performance was intermittent rather than continuous. As such, the subjects’ physiological processes were forced to adapt multiple times, potentially masking the presence of a downward trend in SNS activity such as the one observed in the current study.

### 4.5. ANS Response to Hyperoxia

Lastly, our study demonstrated that a hyperoxic exposure (33% O_2_) following normoxia facilitated parasympathetic activation. When exposed to hyperoxia, the subjects elicited further declines in HR (within healthy levels) and increases in HRV, reflecting the withdrawal of SNS activity and increased parasympathetic dominance. Accumulating evidence shows that the physiological effects of hyperoxia appear paradoxical [45,46]. Studies previously showed that hyperoxia induced vasoconstriction of the cerebral arteries [47] and elicited no effects on cerebral O_2_ availability [48]. Other studies reported that prolonged hyperoxic exposures resulted in mitochondrial damage. Additionally, like the current study, others showed declines in HR and increases in HRV, typically considered an indicator of increased parasympathetic dominance [49]. However, former studies concluded that this response followed the vasoconstriction and increased blood pressure induced by hyperoxia. These counterintuitive findings support the “hyperoxia-hypoxia” paradox, where increased O_2_ from a hyperoxic exposure may induce a hypoxic response that manifests through different mechanisms [50,51,52]. Interestingly though, while the physiological effects of hyperoxia appear negative, several studies previously reported enhanced cognitive outcomes following acute hyperoxic exposures of 30% to 100% O_2_. A 2023 systematic review showed that hyperoxic exposures demonstrated improved memory, attention, problem solving, reaction time, and executive function [53]. Of the 23 studies reviewed, however, none exposed aircrew to flight simulation tasks, very few measured both physiological and cognitive outcomes, and most studies employed designs of “poor quality” [53]. As such, many aspects of using hyperoxia as a strategy for counteracting hypoxic symptoms or enhancing cognition remain unclear.

### 4.6. In-Flight Physiological Monitoring and Influential Factors

**Figure 5:**
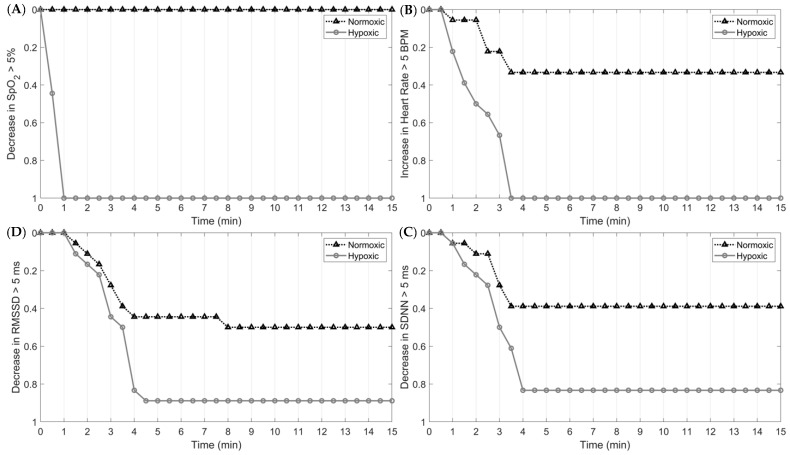
Survival analysis of time to ANS responses at the start of epoch 1 between hypoxic and normoxic exposures: (**A**) >5% change in SpO_2_; (**B**) >5 bpm change in HR; (**C**) >5 ms change in SDNN; (**D**) >5 ms change in rMSSD.

**Figure 6:**
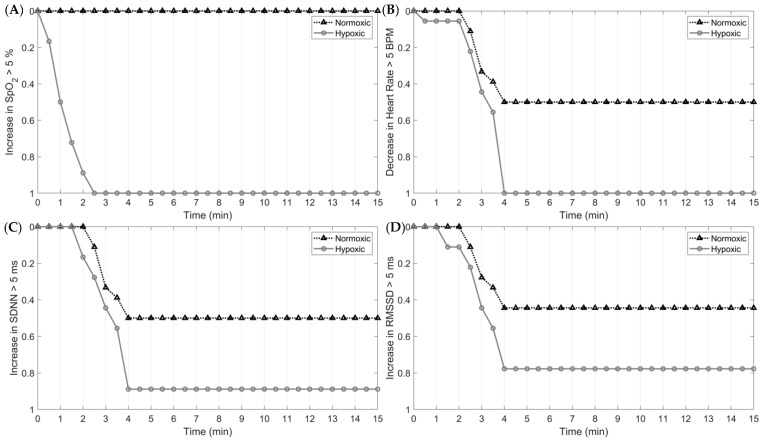
Survival analysis of time to ANS responses at the start of epoch 5 between hypoxic and normoxic exposures: (**A**) >5% change in SpO_2_; (**B**) >5 bpm change in HR; (**C**) >5 ms change in SDNN; (**D**) >5 ms change in rMSSD.

Collectively, the findings of the current study highlight the importance of continuously monitoring physiological parameters during flight operations. Specifically, our study found significant changes to the ANS in response to hypoxia and a high cognitive load. Critically, physiological responses to hypoxia and subsequent normoxia differ in the timing of their response, further supporting the need to monitor multiple physiological parameters. For example, at the onset of hypoxia, the decrease in SpO_2_ occurred rapidly, with 100% of participants eliciting at least a 5% reduction in O_2_ saturation within the first 60 s. Changes in HR and HRV, however, were slower, with 100% of the sample eliciting at least a 5 unit decrease in HR and an increase in rMSSD and SDNN by the fourth min. This same response was shown at the onset of normoxia following the hypoxic exposure; however, the responses were less rapid (Figure 5 and Figure 6). Additionally, our study observed, in parallel with other studies, the high inter-individual variability in the physiological responses to hypoxia, normoxia, and hyperoxia. As such, using a device with the capacity to measure multiple physiological parameters is paramount for optimizing aircrew safety and performance.

In further support, the observations of the current study occurred during a simultaneous exposure to hypoxia and cognitive tasks. This design more accurately reflects the environment to which aircrew are exposed while flying. Designs of former studies often included a “familiarization period” where aircrew could adapt to the hypoxic exposure before performing simulated in-flight tasks. Problematically, aircrew are constantly exposed to a high cognitive load during flight operations and rapidly fluctuate between high and low altitude, with little time for acclimatization. The simultaneous exposures in the current study revealed significantly increased ANS activity, potentially because of higher cognitive load. Additionally, continuous monitoring revealed changes in ANS activity following the first 30 min of the exposures. Taken together, our study shows that the dynamic nature of military aviation and its impact on aircrew demands constant surveillance of their health, ensuring their safety during flight operations.

### 4.7. Strengths and Limitations

The current study possesses strengths and weaknesses that warrant attention. Foremost, this investigation, to the extent of our knowledge, was the first in evaluating the continuous, simultaneous effects of hypoxia and high cognitive load on the ANS. As such, several novel aspects were found and expanded the existing scientific literature optimizing the health and safety of military aircrew. Second, the study design of continuous monitoring of the ANS allowed for greater insight on the nature of the physiological responses to hypoxia and cognitive load, both independently and jointly. Third, the lack of an acclimation period at the onset of the hypoxia trial better reflected the environment to which aircrew are exposed, like rapid changes in altitude during flight. This study also included some limitations. First, the ANS regulates several physiological processes that are also affected by hypoxia, including ventilation, the release of catecholamines, and blood pressure. However, our conclusions cannot extend to these effects, as only changes in HR, HRV, and SpO_2_ were measured in the current study. Second, our study alluded to the possible presence of anxiety at the onset of hypoxia and a high cognitive load; however, this outcome was not directly measured. Third, the smaller sample size precluded the authors from evaluating the influence of important demographic characteristics like age, sex, race/ethnicity, and military occupation.

## 5. Conclusions

In conclusion, the current study demonstrated the heightened activity of the ANS when independently and simultaneously exposed to hypoxia and normoxia. Specifically, we found that within 4 min of the exposure to hypoxia (14% O_2_) and cognitive load, 100% of subjects elicited increases in HR (≥5 bpm) and decreases in HRV, indicating activation of the SNS system. On the other hand, only 20% of the subjects reached this equivalent threshold when normoxic, suggesting that a stronger sympathetic response occurred consequent to hypoxia compared to cognitive load. Following 30 min, both the hypoxic and normoxic subjects appeared to “settle in”, with slight decreases and a near return to baseline for HR and HRV, respectively. Interestingly, the order in which the subjects were exposed to hypoxia (first or second trial) seemed to influence the ANS response such that those receiving hypoxia for the first trial exhibited a more exaggerated response, possibly suggesting an influence of anxiety. Lastly, this study observed that the timing of the physiological responses to hypoxia and cognitive load differed such that at the onset of hypoxia, SpO_2_ responded first, followed by HR and HRV. Conversely, at the onset of normoxia, HR and HRV responded first, followed by SpO_2_. The significant and differential responses of the ANS to the simultaneous exposure of hypoxia and cognitive load strongly suggest that multiple physiological parameters should be continuously monitored during flight operations. For future investigations, we recommend that studies include various levels and orders of hypoxic/hyperoxic/normoxic and cognitive load exposures and measures of anxiety (e.g., cortisol and electromyography), respiration, and brain chemistry (e.g., brain-derived neurotrophic factor and vascular endothelial growth factor). Additionally, larger studies are encouraged to allow for a more in-depth understanding of the ANS responses to varying O_2_ levels and cognitive load, such as the influence of age, sex, military occupation, etc.

## Figures and Tables

**Table 1:** Within-group differences in HR, HRV, and SpO_2_ in response to hypoxia, normoxia, and hyperoxia.

	Hypoxic–Normoxic		Normoxic–Hyperoxic	
	Within-Group Differences		Within-Group Differences	
	Mean (SD)	(*p*-Value)	Mean (SD)	*p*-Value
**Start Epoch 1**		**Epoch 1 vs. 2**		**Epoch 1 vs. 2**
Heart Rate (bpm)	73.5 (9.0)	+6.9 (*p* = 0.002)	68.5 (10.3)	+4.5 (*p* = 0.06)
rMSSD (ms)	38.0 (12.7)	−9.7 (*p* = 0.003)	45.1 (14.5)	−6.4 (*p* = 0.09)
SDNN (ms)	44.3 (14.8)	−11.3 (*p* = 0.003)	52.5 (16.9)	−7.4 (*p* = 0.08)
SpO_2_ (%)	97.7 (2.4)	−8.4 (*p* <0.0001)	98.6 (1.5)	−0.7 (*p* = 0.05)
**Start Epoch 2**		**Epoch 2 vs. 3**		**Epoch 2 vs. 3**
Heart Rate (bpm)	80.4 (10.6)	−0.9 (*p* = 0.68)	73.0 (10.4)	−1.7 (*p* = 0.49)
rMSSD (ms)	28.3 (15.0)	+1.2 (*p* = 0.73)	38.7 (14.6)	+2.4 (*p* = 0.54)
SDNN (ms)	33.0 (17.5)	+1.4 (*p* = 0.72)	45.1 (17.0)	+2.8 (*p* = 0.54)
SpO_2_ (%)	89.3 (4.1)	−0.2 (*p* = 0.19)	97.9 (1.7)	+0.1 (*p* = 0.81)
**Start Epoch 3**		**Epoch 3 vs. 4**		**Epoch 3 vs. 4**
Heart Rate (bpm)	79.5 (9.3)	−0.1 (*p* = 0.92)	71.3 (11.3)	−0.3 (*p* = 0.90)
rMSSD (ms)	29.5 (13.1)	+0.2 (*p* = 0.96)	41.1 (15.9)	+0.4 (*p* = 0.94)
SDNN (ms)	34.4 (15.2)	+0.2 (*p* = 0.96)	47.9 (18.5)	+1.4 (*p* = 0.94)
SpO_2_ (%)	89.1 (4.0)	+0.6 (*p* = 0.29)	98.0 (1.6)	+0.4 (*p* = 0.28)
**Start Epoch 4**		**Epoch 4 vs. 5**		**Epoch 4 vs. 5**
Heart Rate (bpm)	79.4 (9.4)	−2.3 (*p* = 0.32)	71.0 (11.1)	−0.4 (*p* = 0.87)
rMSSD (ms)	29.7 (13.3)	+3.2 (*p* = 0.36)	41.5 (15.6)	+0.6 (*p* = 0.77)
SDNN (ms)	34.6 (15.4)	+3.7 (*p* = 0.35)	48.3 (18.2)	+0.7 (*p* = 0.83)
SpO_2_ (%)	89.7 (5.3)	+0.2 (*p* = 0.038)	98.4 (1.9)	+0.4 (*p* = 0.32)
**Start Epoch 5**	Start Normoxia	**Epoch 5 vs. End**	Start Hyperoxia	**Epoch 5 vs. End**
Heart Rate (bpm)	77.1 (11.3)	+2.3 (*p* = 0.13)	70.6 (11.8)	−3.8 (*p* = 0.24)
rMSSD (ms)	32.9 (15.9)	+5.2 (*p* = 0.16)	42.1 (16.6)	+5.3 (*p* = 0.26)
SDNN (ms)	38.3 (18.5)	+6.1 (*p* = 0.16)	49.0 (19.3)	+6.1 (*p* = 0.26)
SpO_2_ (%)	89.9 (4.4)	+8.9 (*p* < 0.0001)	98.0 (1.9)	+1.3 (*p* = 0.003)
**End Exercise**		**End vs. Start**		**End vs. Start**
Heart Rate (bpm)	73.4 (10.2)	−0.1 (*p* = 0.67)	66.8 (13.9)	−1.7 (*p* = 0.59)
rMSSD (ms)	38.1 (14.4)	+0.1 (*p* = 0.74)	47.4 (19.5)	+2.3 (*p* = 0.64)
SDNN (ms)	44.4 (16.7)	+0.1 (*p* = 0.78)	55.1 (22.7)	+2.6 (*p* = 0.63)
SpO_2_ (%)	98.8 (1.1)	+1.1 (*p* = 0.0003)	99.3 (1.3)	+0.7 (*p* = 0.22)

**Table 2:** Between-group differences in HR, HRV, and SpO_2_ in response to hypoxia, normoxia, and hyperoxia.

	Hypoxia vs. Normoxia	
	Mean Difference	*p*-Value
**Start Epoch 1**		
Heart Rate (bpm)	5.0	*p* = 0.03
rMSSD (ms)	−7.1	*p* = 0.03
SDNN (ms)	−8.2	*p* = 0.03
SpO_2_ (%)	−1.4	*p* = 0.002
**Start Epoch 2**		
Heart Rate (bpm)	7.4	*p* = 0.004
rMSSD (ms)	−10.4	*p* = 0.004
SDNN (ms)	−5.8	*p* = 0.004
SpO_2_ (%)	−7.7	*p* < 0.0001
**Start Epoch 3**		
Heart Rate (bpm)	8.2	*p* = 0.001
rMSSD (ms)	−11.6	*p* = 0.001
SDNN (ms)	−6.8	*p* = 0.001
SpO_2_ (%)	−8.7	*p* < 0.0001
**Start Epoch 4**		
Heart Rate (bpm)	8.3	*p* = 0.001
rMSSD (ms)	−11.8	*p* = 0.001
SDNN (ms)	−6.9	*p* = 0.001
SpO_2_ (%)	−9.9	*p* < 0.0001
**Start Epoch 5**	**Normoxia vs. Hyperoxia**	
Heart Rate (bpm)	6.5	*p* = 0.02
rMSSD (ms)	−9.2	*p* = 0.02
SDNN (ms)	−3.8	*p* = 0.02
SpO_2_ (%)	−7.9	*p* < 0.0001
**End Exercise**		
Heart Rate (bpm)	6.5	*p* = 0.03
rMSSD (ms)	−9.2	*p* = 0.03
SDNN (ms)	−3.0	*p* = 0.03
SpO_2_ (%)	0.3	*p* = 0.50

## Data Availability

Data may be provided upon request.
